# Control of Specific/Nonspecific Protein Adsorption: Functionalization of Polyelectrolyte Multilayer Films as a Potential Coating for Biosensors

**DOI:** 10.3390/ma14247629

**Published:** 2021-12-11

**Authors:** Tomasz Kruk, Monika Bzowska, Alicja Hinz, Michał Szuwarzyński, Krzysztof Szczepanowicz

**Affiliations:** 1 Jerzy Haber Institute of Catalysis and Surface Chemistry, Polish Academy of Sciences, Niezapominajek 8, PL-30239 Krakow, Poland; 2 Department of Cell Biochemistry, Faculty of Biochemistry, Biophysics and Biotechnology, Jagiellonian University, PL-30348 Krakow, Poland; monika.bzowska@uj.edu.pl (M.B.); alicja.karabasz@uj.edu.pl (A.H.); 3 Academic Centre for Materials and Nanotechnology, AGH University of Science and Technology, Mickiewicza 30, PL-30059 Krakow, Poland; szuwarzy@agh.edu.pl

**Keywords:** polyelectrolyte multilayers, poly (ethylene glycol), protein-resistant surfaces, protein adsorption, specific adsorption

## Abstract

Control of nonspecific/specific protein adsorption is the main goal in the design of novel biomaterials, implants, drug delivery systems, and sensors. The specific functionalization of biomaterials can be achieved by proper surface modification. One of the important strategies is covering the materials with functional coatings. Therefore, our work aimed to functionalize multilayer coating to control nonspecific/specific protein adsorption. The polyelectrolyte coating was formed using a layer-by-layer technique (LbL) with biocompatible polyelectrolytes poly-L-lysine hydrobromide (PLL) and poly-L-glutamic acid (PGA). Nonspecific protein adsorption was minimized/eliminated by pegylation of multilayer films, which was achieved by adsorption of pegylated polycations (PLL-g-PEG). The influence of poly (ethylene glycol) chain length on eliminating nonspecific protein adsorption was confirmed. Moreover, to achieve specific protein adsorption, the multilayer film was also functionalized by immobilization of antibodies via a streptavidin bridge. The functional coatings were tested, and the adsorption of the following proteins confirmed the ability to control nonspecific/specific adsorption: human serum albumin (HSA), fibrinogen (FIB), fetal bovine serum (FBS), carcinoembryonic antigen human (CEA) monitored by quartz crystal microbalance with dissipation (QCM-D). AFM imaging of unmodified and modified multilayer surfaces was also performed. Functional multilayer films are believed to have the potential as a novel platform for biotechnological applications, such as biosensors and nanocarriers for drug delivery systems.

## 1. Introduction

Control of specific/nonspecific protein adsorption is one of the main challenges in designing novel biomaterials, such as implants, sensors, or drug delivery systems. It is expected that such biomaterials will likely be in direct contact with biofluids, such as blood or serum [1]. The presence of the components such as blood cells, lipoproteins, plasma proteins, peptides, and their nonspecific adsorption (called fouling or biofouling) is the most critical problem for practical applications of biomaterials besides cost considerations [2]. The novel biomaterials are very promising when tested in a simple, well-controlled environment; however, their effectiveness is not yet satisfied when tested in real-life environments with much more complex chemistries. In the case of, e.g., biosensors, the operation is based on specific interactions (specific adsorption). The specific adsorption of proteins to biomaterials results mainly from noncovalent interactions between the system’s biomolecules and the biomaterial’s molecules. These bonds are favored by the appropriate steric arrangement of proteins and biomaterial molecules, the so-called system complementarity. Such bonds are most often formed in the systems: ligand–receptor, antibody–antigen, enzyme–substrate, and are the basis for the functioning of most biosensors. However, these interactions can be effectively disturbed by nonspecific adsorption of other proteins in the system. The adsorbed surface layer of other proteins caused by excessive biofouling not only blocks access to recognition ligands, e.g., antibodies, preventing quantification, or even detection of analytes, but also overwhelms the transduction process in biosensors by generating “false positive” signals [2]. Nonspecific adsorption, also based on noncovalent interactions, is independent of the complementarity of the protein–biomaterial system. It depends mainly on the physicochemical properties of the protein and biomaterial and the environmental conditions (pH, ionic strength, temperature). Despite decades of research on “protein resistant surfaces”, biofouling is still a main limiting factor in the reliable performance of biomaterials. A commonly used method to reduce nonspecific adsorption is a surface modification with inert, hydrophilic polymers. Such polymers contain hydrogen bond acceptors and do not have hydrogen bond donors.

The most popular compounds used for this purpose are polysaccharides, including dextran, heparin, polyacrylates, and phosphorylcholine, and polyethylene glycol (PEG), also referred to as polyethylene oxide (PEO) [2,3]. Fast and sensitive determination of biologically active compounds is very important in biomedical diagnostics, the food and beverage industry, and environmental analysis. Conducting polymers (CPs) and composites with different nanomaterials have received attention. CPs can be applied in the design of sensors and biosensors on account of numerous technological advantages, e.g., the immobilization of biological recognition elements (based on enzymes, ssDNA, antibodies (Ab), receptors, and other biological proteins). The ability to design molecularly imprinted polymers can form artificial structures, which might replace some natural biological structures, e.g., DNA aptamers or biological-recognition-exhibiting proteins. The sensors based on CPs can give a high sensitivity, a short response time, and monitor at room temperature. The conducting polymers might be applied in the design of molecularly imprinted polymers, which are cheap and they might replace natural recognition elements. One of the main goals in the improvement of CP-based biosensors is related to the stability of the analytical signal. The biocompatibility aspects of conducting polymers encourage one to apply these polymers in the design of implantable biofuel cells, which can serve as power sources of some implantable biomedical devices [4,5,6,7,8,9,10].

PEG is one of the most promising materials due to its biocompatibility, low toxicity, and immunogenicity, and high efficiency in the process of reducing nonspecific protein adsorption [11,12]. The ability of PEG-modified coatings to eliminate nonspecific protein adsorption has been proved, presenting the meaning of chain length and brush density [13,14,15,16,17,18]. The PEG chain length should be sufficient to screen protein–substrate interactions, and the brush chain density should be enough to block diffusion through this PEG layer. The PEG monodispersity also influences on antifouling propertiers [19]. A useful method to immobilize PEG chains on the charged surface is to use pegylated polyelectrolytes, such as poly (l-lysine) (PLL), poly (l-glutamic acid) (PGA), or poly (acrylic acid) (PAA) [20,21,22,23,24]. Such polyelectrolytes with grafted PEG chains are deposited by electrostatic interactions between the charged backbone and the oppositely charged surface [25]. The layer-by-layer (LbL) technique of electrostatic self-assembly of charged nano-objects has been proved to be a versatile technique of surface modification and preparation of tailored functional coatings for a wide range of applications, including biomedicine [26,27,28,29,30,31,32,33,34,35,36]. The method is based on the sequential adsorption of the oppositely charged species on charged surfaces. Protein adsorption on various polyelectrolyte coatings formed through the layer-by-layer technique showed that electrostatic interactions govern this process [37,38,39,40]. The proteins strongly interact with the polyelectrolyte film, whatever the sign of the charges of both the multilayer and the protein. It was shown that electrostatic forces dominated the interaction between proteins and the multilayer film. In the case of the proteins and the outermost PE layer having equal charge, a dense monolayer of adsorbed proteins is created. If the charges are opposite, then a thick protein layer is noticed [40]. Since nonspecific protein adsorption is the most critical problem for practical applications of multilayers as a coating of biosensors or micro- and nano-capsules, it should be minimized or even eliminated to allow the proper operation of such biomaterials. Moreover, further functionalization for specific recognition or specific interaction may allow better sensitivity and activity of developed biomaterials.

PLL-g-PEG is a well-known pegylated polyaminoacid that is used to repeal proteins when adsorbed on an oppositely charged surface. Heuberger et al. showed that multilayers formed with synthetic polyelectrolytes PAH and PSS deposited onto flat silica or microcapsules and functionalized by the PLL-g-PEG effectively eliminate nonspecific protein adsorption. Moreover, they showed that biotin-modified PLL-g-PEG can be used as a model system to induce bispecific streptavidin bindings [41,42]. For the formation of protein-resistant polyelectrolyte coatings, pegylated copolymers, such as PGA-g-PEG, were deposited on the polyelectrolyte multilayers as the external layer. That pegylated polyelectrolyte coating efficiently repels proteins [21,37,43,44]. Since, in our previous work, we were focused on the detailed investigation of multilayered polyaminoacids films modified with PGA-g-PEG, here, we focused on the fundamental study concerning the influence of PEG chain length of commercially available PLL-g-PEG on antiadhesive properties of such pegylated polyaminoacids multilayer coatings. Moreover, such pegylated with PLL-g-PEG coatings were additionally functionalized by immobilization of biotinylated antibodies as a receptor for specific protein adsorption. QCM-D, as well as AFM techniques, were applied to control polyaminoacids multilayer film formation, its pegylation, and functionalization, as well as further protein adsorption. The human serum albumin (HSA), fibrinogen (FIB), and full blood serum (FBS) were chosen because of their fundamental meanings (HSA and FIB are the main proteins in human blood plasma with high adsorption properties).

## 2. Materials and Methods

### 2.1. Chemicals

Proteins: human serum albumin (HSA), fibrinogen (FIB), carcinoembryonic antigen human (CEA), and streptavidin (STREPT) from Streptomyces avidinii were purchased from Sigma-Aldrich, Poznan, Poland, while fetal bovine serum (FBS) and Dulbecco’s modified Eagle medium (DMEM) were from Gibco (Thermo Fischer Scientific, Waltham, MA, USA). Polyelectrolytes: poly-L-lysine hydrobromide (PLL, MW ~15,000 to 30,000) and poly-L-glutamic acid sodium salt (PGA, MW ~15,000 to 50,000) were purchased from Sigma-Aldrich, Poznan, Poland, while pegylated polyelectrolytes PLL-g-PEG2k PLL(MW 20 kDa)-g(3.5)-PEG(MW 2 kDa), PLL-g-PEG5k PLL(MW 20 kDa)-g(3,5)-PEG(MW 5 kDa), and PLL-g-PEG-BIO PLL(MW 20 KDa)-g (3.5)-PEG(MW 2 KDa)/PEGbio(MW 3.4 KDa 50%) were obtained from SuSoS AG, Dübendorf, Switzerland. Sodium chloride (NaCl) was purchased from Sigma-Aldrich, Poznan, Poland, while sulphuric acid 96% (H_2_SO_4_) analytical grade and hydrogen peroxide (30%) were purchased from Avantor Performance Materials, Gliwice, Poland. All the materials were used as received without further purification. Distilled water used in all experiments was obtained with the three-stage Millipore Direct-Q 5UV purification system.

### 2.2. Production and Biochemical Modification of Anti-CEA Antibody. Animals and Cells Used in Studies

A six-week-old BALB/c female mouse was provided by the Charles River Laboratories (supplier in Poland—AnimaLAB, Poznań, Poland). The mouse was housed under controlled conditions and provided with food and water ad libitum. According to Polish law, all animal procedures were performed specifically to the Act on the Protection of Animals used for Scientific or Educational Purposes (D20150266L), which implements the European Parliament’s Directive and the Council (2010/63/EU). All procedures agreed with the Institutional Animal Care and Use Committee (IACUC) guidelines and were approved by the 2nd Local IACUC in Kraków.

Mouse myeloma cell line SP2/0-Ag14 (ATCC CRL-1581) was purchased in American Type Culture Collections (Manassas, VA, USA). Mouse hybridoma cell line B2G8 producing monoclonal antibody specific to CEA was obtained in the Department of Cell Biochemistry, Faculty of Biochemistry, Biophysics and Biotechnology, Jagiellonian University (Krakow, Poland). 

### 2.3. Reagents Used for Antibody Production

Materials used for cell culture (DMEM, fetal bovine serum) were purchased in LONZA (Basel, Switzerland). Hipoxanthine–aminopterin–thymidine (HAT) and hipoxanthine–thymidine (HT) media supplements, HybriMAX PEG were from Sigma Aldrich.

### 2.4. Production, Purification, and Biotinylation of a Murine Monoclonal Antibody Specific to CEA

Mouse monoclonal antibodies specific to CEA protein were obtained using hybridoma technology, according to the standard procedure. Briefly, a six-week-old Balb/C mouse was injected intraperitoneally with 100 µg of antigen (CEA, Sigma Aldrich, St. Louis, MO, USA) diluted in PBS and mixed 1:1 with complete Freund’s adjuvant (Sigma Aldrich, St. Louis, MO, USA). Subsequent immunizations were performed with 50µg of antigen mixed with incomplete Freund’s adjuvant (Sigma Aldrich, St. Louis, MO, USA). After administering appropriate anesthetic (ketamine and xylazine), the animal was euthanized, the spleen was isolated, and splenocytes were immortalized by fusion with mouse myeloma SP2/0-Ag14 cells (ATCC CRL-1581). After fusion, hybridoma cells were cultured in a selection medium (DMEM, 10% FBS, HAT) and tested using enzyme-linked immunosorbent assay (ELISA) to identify clones that produce antibodies specific to CEA. Hybridoma cells clone B2G8 producing monoclonal antibodies specific to CEA were grown according to the manufacturer’s instruction in CELLine bioreactors (INTEGRA Bioscences AG) to produce large amounts of monoclonal antibodies. Monoclonal antibodies B2G8 were then purified from the culture medium by affinity chromatography on protein L according to the protocol provided by the manufacturer (Thermo Fisher Scientific, Waltham, MA, USA), and then immediately dialyzed into sterile PBS. The concentration of purified antibodies was measured using the bicinchoninic acid method (Sigma Aldrich, St. Louis, MO, USA). Prior biotinylation antibodies were dialyzed to sterile carbonate buffer (0.1 M, pH = 9) and conjugated with EZ-Link NHS-LC-Biotin (Thermo Scientific, Waltham, MA, USA) according to the standard protocol. Efficient biotinylation of antibodies was confirmed in an ELISA test using plates coated with CEA. Streptavidin conjugated to horseradish peroxidase (Sigma Aldrich, St. Louis, MO, USA) was used to detect B2G8 to CEA antigen immobilized on the ELISA plate. 

### 2.5. Formation of Functional Polyelectrolyte Films

**Figure 1:**
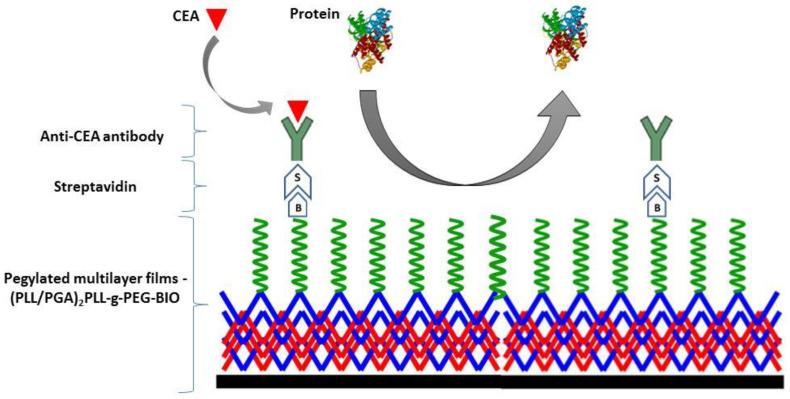
Schematic representation of multilayer functional films.

The polyelectrolyte multilayer films were created via the layer-by-layer (LbL) technique. The sequential depositions were conducted on QCM-D sensors; the processes went on until dissipation and frequency signals reached constant values and were followed by rinsing by the buffer. The process of multilayer preparation was started with polycation—PLL (0.1 g/L in 0.015 M NaCl, rinsing 0.015 M NaCl). Secondly, a method was conducted for the negatively charged polyelectrolyte—PGA (0.1 g/L in 0.015 M NaCl, rinsing 0.015 M NaCl). The procedure was repeated until the four layers were formed. Such (PLL/PGA)_2_ negatively charged polyelectrolyte multilayers (PGA-ended) were pegylated by the adsorption of the pegylated polycations PLL-g-PEG and PLL-g-PEG-BIO, respectively (0.1 g/L in 0.015 M NaCl, rinsing 0.015 M NaCl). The pegylated multilayers with biotin groups were further functionalized by the attachment of streptavidin (0.01 g/L in 0.015 M NaCl, rinsing 0.015 M NaCl) and biotinylated antibody, i.e., biotinylated anti-CEA (0.01 g/L in 0.015 M NaCl, rinsing 0.015 M NaCl). Schematic representation of multilayer functional films is presented in Figure 1.

### 2.6. Specific/Nonspecific Protein Adsorption Tests

Specific/nonspecific protein adsorption tests were performed on QCM-D sensors previously functionalized with proper multilayer film. For nonspecific adsorption, the attachment of human serum albumin (HSA), fibrinogen (FIB) (in 0.015 M NaCl), as well as full fetal bovine serum (FBS, in DMEM) was subsequently measured by QCM-D, while specific adsorption was tested by attachment of carcinoembryonic antigen human to anti-CEA functionalized surface. 

### 2.7. QCM-D Studies

The preparation of PEMs with pegylated copolymers using the LbL method, as well as protein attachments, was studied by the QCM-D QSense E4 system (Biolin Scientific, Gothenburg, Sweden) according to the protocol described previously [43]. The adsorption process was performed in the flow cell on quartz sensors (14 mm diameter Q-Sensor QSX 301 Gold, Q-Sense) covered with gold electrodes (resonance frequency of 4.95 MHz ± 50 kHz). The temperature during adsorption was set up at 22 °C; flow velocity was 0.3 mL/min. The adsorbed layers were rigid (dissipation was less than 1 × 10^− 6^ per 10 Hz of the frequency shift), the mass of deposited film was received with the Sauerbrey equation [45]. The calculations were performed with Qtools 3 software (QSense, Biolin Scientific, Gothenburg, Sweden). The adsorbed mass was the average of the results from four QCM-D cells. The experimental error was less than 10% of the measured mass.

### 2.8. Atomic Force Microscopy Studies

Atomic force microscope (AFM) images were obtained with Dimension Icon XR atomic force microscope (Bruker, Santa Barbara, CA, USA) working in the water in the PeakForce Tapping (PFT) mode using standard silicon cantilevers of nominal spring constant of 0.7 N/m and triangular geometry tip with a nominal tip radius of 2 nm.

## 3. Results

In our previous work [43], we tested PGA-g-PEG (pegylated polyanion) surface-treatment technology as an antiadhesive coating. The adsorption of proteins (HSA, FIB, and FBS) onto pegylated polyaminoacid multilayers were decreased in relation to the multilayered film without a pegylated external layer. The amount of adsorbed proteins decreased with the increasing molecular weight and surface density of PEG chains. For PGA-g-PEG g > 30% and PEG 5000, very low levels of protein deposition were obtained. Here, we focused on analog approaches with pegylated polycation PLL-g-PEG, commercially available poly-L-lysine with grafted PEG chains. Obtained results were compared with our previous results for pegylated polyanion; moreover, here, we functionalized such a system for specific protein adsorption based on antibody–antigen interaction. 

### 3.1. Elimination of Nonspecific Protein Adsorption

**Figure 2:**
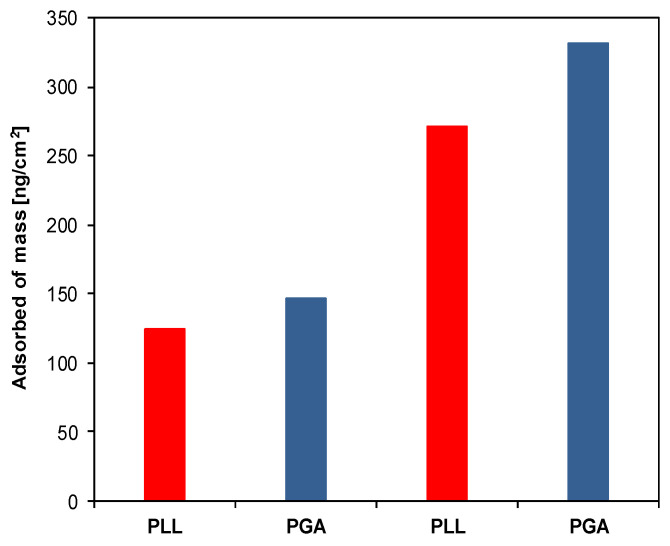
QCM controlling of the creation of (PLL/PGA)_2_ films. The mass (ng/cm^2^) of deposited layers was calculated with the Sauerbrey equation [36].

The preparation of protein-resistant layers is the main challenge in the formation of new nanomaterials. Such nonspecific protein adsorption can disturb the application of biomaterials. The protein-resistant coating was formed by the layer-by-layer technique using biocompatible polyelectrolytes PLL, PGA, and pegylated-PLL. The formation of multilayer film was investigated in situ by the QCM-D. The gold surface (sensor) in the experimental conditions (pH ≈ 6–8) is negatively charged [46]; therefore, the first polyelectrolyte, which was introduced into the QCM cell, was polycation (PLL). In the next step, the rinsing solution (0.015 M NaCl) was brought into the cell. When the signal of QCM-D was stable, the PGA solution was brought into the cell, and the polyanion deposition step was conducted. This process was repeated until four polyelectrolyte layers were formed. The growth of the film mass for the PLL/PGA multilayer formation on the QCM-D sensor is shown in Figure 2. 

**Figure 3:**
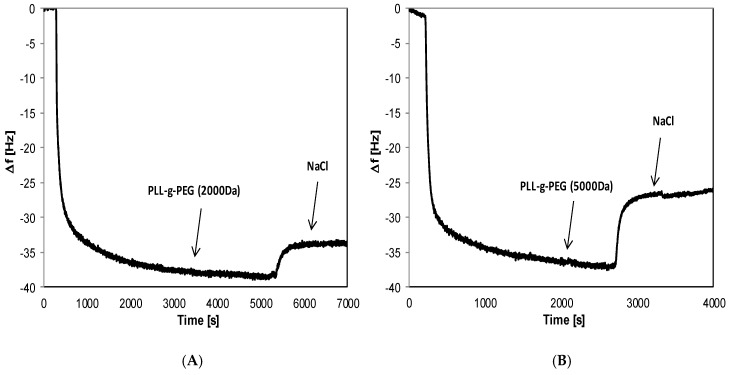
The example of the QCM-D frequency signal changes upon adsorption and rinsing solution (0.015 M NaCl) of (**A**)—PLL-g-PEG(2000 Da) and (**B**)—PLL-g-PEG(5000 Da) on (PLL/PGA)_2_ layer.

It can be noticed that the increase in the polyelectrolyte film mass is exponential for (PLL/PGA)_2_ adsorption, which is in agreement with the previous work for this kind of PEM [39,47]. The negatively charged films (PLL/PGA)_2_ were pegylated by the adsorption of the positively charged copolymers PLL-g-PEG2k and PLL-g-PEG5k. The decrease in the frequency of crystal resonance confirmed the spontaneous deposition of pegylated-PLL on top of the negatively charged polyelectrolyte multilayer structures. The typical QCM-D frequency signal changes upon the pegylated copolymer deposition process are shown in Figure 3. 

The adsorption kinetics for both pegylated polyelectrolytes were similar and agreed with the kinetics of adsorption at other surfaces, e.g., various oxides [43]. The adsorption process resulted in the pegylated layer formation with an areal density of approximately 540–700 ng/cm^2^ (cf. Table 1). The obtained mass of deposited copolymers was different and had a connection with the MW of PEG chains. It is worth noticing that the mass of deposited copolymer decreases with the length of PEG chain, and it may be a result of the steric repulsion of longer PEG chains of the cationic copolymer, which shields the electrostatic attraction of the negative external layer of the coating; similar behavior was also observed for PGA-g-PEGs [43].

**Figure 4:**
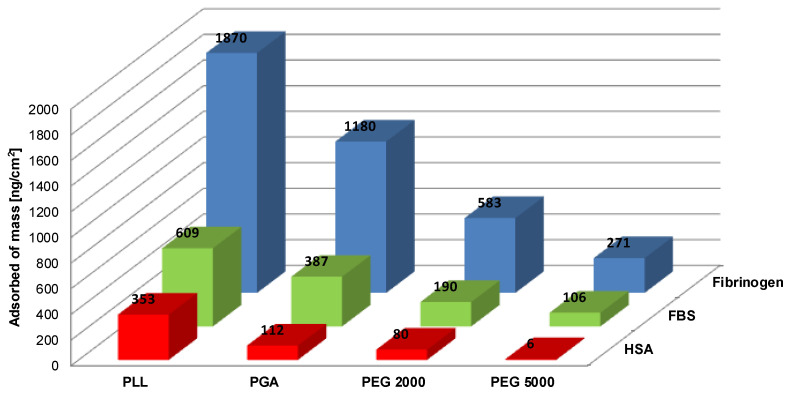
The mass per unit area of adsorbed proteins: HSA, fibrinogen, and FBS on top of tested surfaces ended in PLL, PGA, and different PLL-g-PEG.

Nonspecific protein adsorption tests were performed by QCM-D on the sensors previously functionalized with proper multilayer film. The films finished by positively charged PLL, negatively charged PGA, and two varied pegylated-PLL were studied. For nonspecific adsorption, the attachment of human serum albumin (HSA), fibrinogen (FIB) (both in 0.015 M NaCl), and full fetal bovine serum (FBS, in DMEM) were studied. In advance, deposited FBS (dissolved in DMEM) films were conditioned in DMEM buffer. Figure 4 shows the mass of proteins (HSA, fibrinogen, and FBS) on the top of tested films. 

It seems that deposition of proteins is the highest on the top of the PLL-ended, positively charged polyelectrolyte film, while the adsorption on PGA-finished, negatively charged film led to a lower mass in comparison to the one at the positively charged film. The results show that the adsorption process of proteins is driven by electrostatic forces. The comparable results of protein adsorption on polyelectrolyte multilayers were achieved previously [22,39,43,47].

In comparison with nonpegylated films, pegylated ones significantly reduce nonspecific protein adsorption. The results showed the role of the thickness of PEG layer in shielding the surface charge. The amounts of deposited proteins decreased with the increasing molecular weight of PEG chains. It can be stated that, for a higher molecular weight of PEG chains (5000 Da), the most effective process of reducing the adsorption of the analyzed proteins was observed. The results are comparable for an analogous approach using pegylated polyelectrolytes: polycations PLL-g-PEG [21,37,44] on TiO_2_, Nb_2_O_5,_ or Si_0.4_Ti_0.6_O_2_ surfaces. When we compare our previous results for PGA-g-PEG with those presented here for PLL-g-PEG, a significant impact of the type of polyelectrolyte backbone in pegylated copolymers can be observed. For anionic PGA-g-PEG, more effective elimination of nonspecific adsorption is observed compared to cationic PLL-g-PEG. It can be the effect of various conformations of macromolecules after adsorption at surfaces when not only trails, but also loops and tails are available. Such structures formed with a positively charged backbone of PLL-g-PEG copolymers may result in higher adsorption of negatively charged, spacious compared with negatively charged, backbone PGA-g-PEG [43].

### 3.2. Specific Protein Interaction

**Figure 5:**
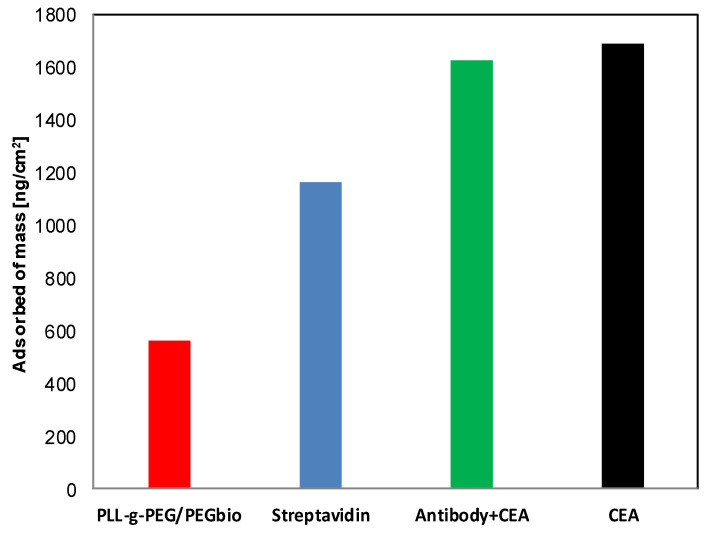
The mass per unit area of adsorbed PLL-g-PEG/PEGbio and proteins on top of tested surfaces ended in (PLL/PGA)_2_.

The nonspecific protein adsorption of other proteins in the system may disturb specific protein adsorption based on antibody–antigen interaction. Therefore, the first step of the functionalization is forming multilayer protein-resistant coatings, as presented in the previous paragraph. Here, such a coating formed by the layer-by-layer technique using biocompatible polyelectrolytes PLL, PGA, and pegylated-PLL was additionally functionalized by immobilization of the selected antibody, providing the ability of specific protein adsorption/interaction. For so-called “bioconjugation”, a simple approach with a streptavidin bridge was selected [48]. Such an approach allows proper immobilization of biotinylated antibodies to the biotinylated surface [48]; it is one of the available, well-accepted methods of immobilization of antibodies that do not affect their activity and should ensure the appropriate spatial orientation of the ligand. For that reason, commercially available pegylated-PLL with additional biotin groups at the end of PEG chains was used. Results presented in the previous paragraph indicate that the higher molecular weight of PEG chains provides the most effective reduction in nonspecific protein adsorption; however, in terms of weight of PEG chains, there was only one commercially available option (PLL(20)-g(3.5)-PEG(2)/PEG(3.4)-biotin(50%)). Despite that not being optimal (the available molecular weight of PEG was 2/3.4 kDa), the additional synergistic effect from immobilized antibodies may be achieved. To the functional biotin groups located at the end of PEG chains, streptavidin was attached, followed by biotinylated antibody anti-CEA. The process was monitored by QCM-D, and the frequency signal changes and mass changes upon the deposition process are shown in Figure 5. 

Based on the presented results, it can be stated that streptavidin binds to PLL-g-PEG-BIO (604 ng/cm^2^) and the biotinylated antibody binds to the streptavidin (464 ng/cm^2^). 

**Figure 6:**
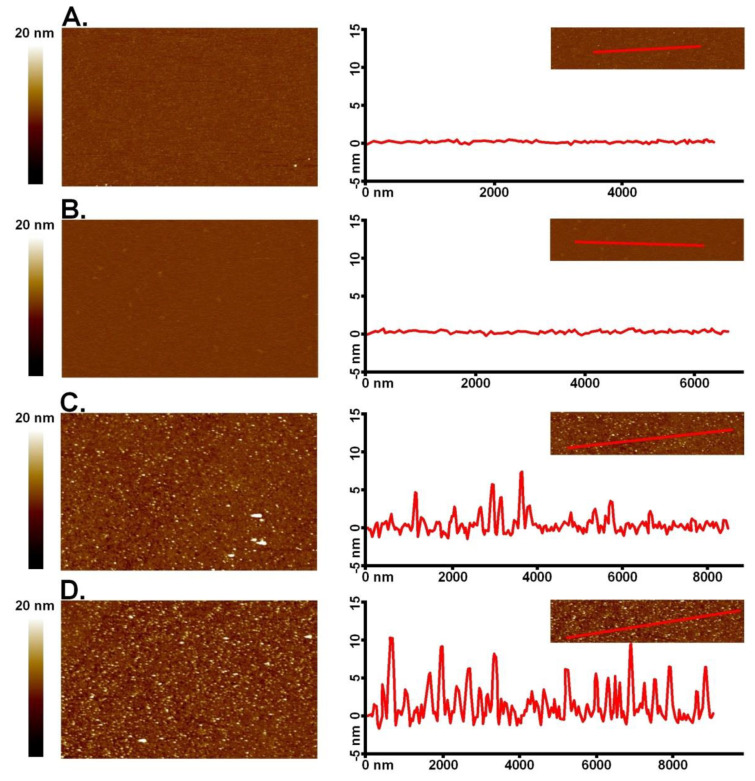
AFM images with corresponding cross-sections of (**A**) (PLL/PGA)_2_ multilayer film, (**B**) PLL-g-PEG-BIO on (PLL/PGA)_2_ layers, (**C**) streptavidin on polyelectrolyte film (PLL/PGA)_2_ with PLL-g-PEG-BIO, and (**D**) (PLL/PGA)_2_/PLL-g-PEG-BIO/and the biotinylated antibody binds to the streptavidin.

The obtained results were also confirmed by imaging the surfaces of polyelectrolyte multilayers using AFM. Figure 6A,B shows the surfaces of films for (PLL/PGA)_2_ and (PLL/PGA)_2_ with an external PLL-g-PEG-BIO layer, respectively. In both cases, it can be seen that the films obtained are flat, with little roughness (RMS), Figure 6A: 0.268 ± 0.004 nm and Figure 6B: 0.287 ± 0.011 nm, a typical tendency for polyelectrolyte multilayers. On the other hand, in the case of films with attached streptavidin to the copolymer with PEG-bio and the antibody to streptavidin, clearly adsorbed proteins can be observed (Figure 6C,D). A significant surface change can also be seen with an increase in the roughness of multilayers film as a result of protein adsorption, Figure 6C: 1.194 ± 0.021 nm and Figure 6D: 1.727 ± 0.036 nm, respectively. The results suggest that the streptavidin binds to PLL-g-PEG-BIO and the biotinylated antibody binds to the streptavidin, which is compatible with the results from QCM-D measurements.

Specific protein adsorption/interaction tests were performed by QCM-D on the gold sensors previously functionalized with proper multilayer film with immobilized anti-CEA antibody by the adsorption of two model proteins: human CEA antigen (specific to anti-CEA antibody) and HSA. Results of such experiments are presented in Table 2. Specific adsorption/interaction can be clearly seen, since the average adsorbed mass of human CEA antigen on antibody-functionalized surface was 60 ng/cm^2^, while HSA does not adsorb at the tested surface. It is worth noticing that nonspecific adsorption of HSA was reduced almost to zero, and a synergistic effect from pegylation and antibody immobilization was achieved. The proposed method of functionalization, however simple, may be a candidate for future applications in both the biomaterial/implant, biosensor areas, and targeted drug delivery systems.

## 4. Conclusions

We tested polyelectrolyte multilayer coating formed with biocompatible polyelectrolytes as functional coatings for controlling specific/nonspecific protein adsorption. The coating was formed by a simple approach, i.e., layer-by-layer technique. Polyelectrolyte multilayer coating was functionalized by pegylation with PLL-g-PEG to eliminate nonspecific protein adsorption and by antibody immobilization to achieve specific protein adsorption/interaction. The adsorption of proteins onto pegylated polyelectrolyte multilayers was decreased in relation to the films without PEG layer, and the amount of proteins depended on the molecular weight of PEG chains; moreover, such a system was additionally functionalized to control protein adsorption/interaction. Biotinylated antibody was attached via a streptavidin bridge. QCM-D measurements confirmed specific adsorption/interaction. The proposed and tested approach with PLL-g-PEG surface-treatment technology may be a candidate for future applications in the biomaterial and biosensor areas to effectively detect specific protein interactions; however, further investigation related to the final application is necessary.

## Figures and Tables

**Table 1:** The areal density of two different adsorbed pegylated copolymers.

Multilayer Structure	Pegylated Polyelectrolyte	Areal Density
(PLL/PGA)_2_PLL-gPEG2k	PLL(20 kDa)-g(3.5)-PEG(2 kDa)	700 ng/cm^2^
(PLL/PGA)_2_PLL-gPEG5k	PLL(20 kDa)-g(3.5)-PEG(5 kDa)	542 ng/cm^2^

**Table 2:** Adsorbed mass of proteins at functionalized surfaces.

Surface	Mass of Proteins (ng/cm^2^)	Mass of Proteins (ng/cm^2^)
	HSA	CEA
(PLL/PGA)_2_PLL-g-PEGBIO	70	180
(PLL/PGA)_2_PLL-g-PEGBIO/ STREPT/BIO-Anti-CEA	~0	60

## Data Availability

Not applicable.

## References

[1] Blaszykowski C., Sheikh S., Thompson M. (2012). Surface chemistry to minimize fouling from blood-based fluids. Chem. Soc. Rev..

[2] Zhang S., Geryak R., Geldmeier J., Kim S., Tsukruk V.V. (2017). Synthesis, Assembly, and Applications of Hybrid Nanostructures for Biosensing. Chem. Rev..

[3] Malmsten M. (1995). Protein Adsorption at Phospholipid Surfaces. J. Colloid Interface Sci..

[4] Ramanavicius S., Ramanavicius A. (2021). Conducting Polymers in the Design of Biosensors and Biofuel Cells. Polymers.

[5] Ramanavicius S., Jagminas A., Ramanavicius A. (2021). Advances in Molecularly Imprinted Polymers Based Affinity Sensors. Polymers.

[6] Spychalska K., Zając D., Baluta S., Halicka K., Cabaj J. (2020). Functional Polymers Structures for (Bio)Sensing Application—A Review. Polymers.

[7] Ramanavicius S., Ramanavicius A. (2021). Charge Transfer and Biocompatibility Aspects in Conducting Polymer-Based Enzymatic Biosensors and Biofuel Cells. Nanomaterials.

[8] Plausinaitis D., Sinkevicius L., Samukaite-Bubniene U., Ratautaite V., Ramanavicius A. (2020). Evaluation of electrochemical quartz crystal microbalance based sensor modified by uric acid-imprinted polypyrrole. Talanta.

[9] Lakard B. (2020). Electrochemical Biosensors Based on Conducting Polymers: A Review. Appl. Sci..

[10] Ramanavicius S., Ramanavicius A. (2020). Progress and Insights in the Application of MXenes as New 2D Nano-Materials Suitable for Biosensors and Biofuel Cell Design. Int. J. Mol. Sci..

[11] Harris J.M. (1992). Poly(Ethylene Glycol) Chemistry: Biotechnical and Biomedical Applications.

[12] Thiele L., Rothen-Rutishauser B., Jilek S., Wunderli-Allenspach H., Merkle H.P., Walter E. (2001). Evaluation of particle uptake in human blood monocyte-derived cells in vitro. Does phagocytosis activity of dendritic cells measure up with macrophages?. J. Control. Release.

[13] Milton Harris J., Chess R.B. (2003). Effect of pegylation on pharmaceuticals. Nat. Rev. Drug Discov..

[14] Vermette P., Meagher L. (2003). Interactions of phospholipid- and poly(ethylene glycol)-modified surfaces with biological systems: Relation to physico-chemical properties and mechanisms. Colloids Surf. B Biointerfaces.

[15] Halperin A. (1999). Polymer brushes that resist adsorption of model proteins: Design parameters. Langmuir.

[16] Zhu B., Eurell T., Gunawan R., Leckband D. (2001). Chain-length dependence of the protein and cell resistance of oligo(ethylene glycol)-terminated self-assembled monolayers on gold. J. Biomed. Mater. Res..

[17] Prime K.L., Whitesides G.M. (1993). Adsorption of proteins onto surfaces containing end-attached oligo(ethylene oxide): A model system using self-assembled monolayers. J. Am. Chem. Soc..

[18] Michel R., Pasche S., Textor M., Castner D.G. (2005). Influence of PEG architecture on protein adsorption and conformation. Langmuir.

[19] Zhang P., Zhang Z., Wang D., Hao J., Cui J. (2020). Monodispersity of Poly(ethylene glycol) Matters for Low-Fouling Coatings. ACS Macro Lett..

[20] Pasche S., Voros J., Griesser H.J., Spencer N.D., Textor M. (2005). Effects of ionic strength and surface charge on protein adsorption at PEGylated surfaces. J. Phys. Chem. B.

[21] Huang N., Michel R., Voros J., Textor M., Hofer R., Rossi A., Elbert D.L., Hubbell J.A., Spencer N.D. (2001). Poly (L-lysine)-g-poly (ethylene glycol) layers on metal oxide surfaces: Surface-analytical characterization and resistance to serum and fibrinogen adsorption. Langmuir.

[22] Boulmedais F., Frisch B., Etienne O., Lavalle P., Picart C., Ogier J., Voegel J., Schaaf P., Egles C. (2004). Polyelectrolyte multilayer films with pegylated polypeptides as a new type of anti-microbial protection for biomaterials. Biomaterials.

[23] Schmolke H., Hartwig S., Klages C. (2011). Poly (acrylic acid)-graft-poly (ethylene glycol) preparation and adsorption on polyelectrolyte multilayers (PEMs) for custom-made antiadhesive surfaces. Phys. Status Solidi.

[24] Klages C., Hartwig S., Schmolke H. (2011). Adsorption of Poly (Acrylic Acid)-Graft-Poly (Ethylene Glycol) on Polyelectrolyte Multilayers. Anonymous Trends in Colloid and Interface Science XXIV.

[25] Wattendorf U., Merkle H.P. (2008). PEGylation as a tool for the biomedical engineering of surface modified microparticles. J. Pharm. Sci..

[26] Seyrek E., Decher G., Matyjaszewski K., Müller M. (2012). Layer-by-Layer Assembly of Multifunctional Hybrid Materials and Nanoscale Devices. Polymer Science: A Comprehensive Reference.

[27] Decher G., Hong J. (1991). Buildup of ultrathin multilayer films by a self-assembly process, 1 consecutive adsorption of anionic and cationic bipolar amphiphiles on charged surfaces. Makromol. Chem. Macromol. Symp..

[28] Zhao S., Caruso F., Dähne L., Decher G., De Geest B.G., Fan J., Feliu N., Gogotsi Y., Hammond P.T., Hersam M.C. (2019). The Future of Layer-by-Layer Assembly: A Tribute to ACS Nano Associate Editor Helmuth Möhwald. ACS Nano.

[29] Iler R.K. (1966). Multilayers of colloidal particles. J. Colloid Interface Sci..

[30] Decher G., Hong J., Schmitt J. (1992). Buildup of ultrathin multilayer films by a self-assembly process: III. Consecutively alternating adsorption of anionic and cationic polyelectrolytes on charged surfaces. Thin Solid Film..

[31] Kruk T., Szczepanowicz K., Kręgiel D., Szyk-Warszynska L., Warszynski P. (2016). Nanostructured multilayer polyelectrolyte films with silver nanoparticles as antibacterial coatings. Colloids Surf. B Biointerfaces.

[32] Decher G., Schlenoff J.B. (2006). Multilayer Thin Films: Sequential Assembly of Nanocomposite Materials.

[33] Richardson J.J., Bjornmalm M., Caruso F. (2015). Multilayer assembly. Technology-driven layer-by-layer assembly of nanofilms. Science.

[34] Sukhorukov G.B., Donath E., Lichtenfeld H., Knippel E., Knippel M., Budde A., Möhwald H. (1998). Layer-by-layer self assembly of polyelectrolytes on colloidal particles. Colloids Surf. Physicochem. Eng. Asp..

[35] Karabasz A., Bzowska M., Łukasiewicz S., Bereta J., Szczepanowicz K. (2014). Cytotoxic activity of paclitaxel incorporated into polyelectrolyte nanocapsules. J. Nanoparticle Res..

[36] Bazylinska U., Skrzela R., Piotrowski M., Szczepanowicz K., Warszynski P., Wilk K.A. (2012). Influence of dicephalic ionic surfactant interactions with oppositely charged polyelectrolyte upon the in vitro dye release from oil core nanocapsules. Bioelectrochemistry.

[37] Kenausis G.L., Vörös J., Elbert D.L., Huang N., Hofer R., Ruiz-Taylor L., Textor M., Hubbell J.A., Spencer N.D. (2000). Poly (L-lysine)-g-poly (ethylene glycol) layers on metal oxide surfaces: Attachment mechanism and effects of polymer architecture on resistance to protein adsorption. J. Phys. Chem. B.

[38] Yang J.M., Tsai R., Hsu C. (2016). Protein adsorption on polyanion/polycation layer-by-layer assembled polyelectrolyte films. Colloids Surf. B Biointerfaces.

[39] Baggerman J., Smulders M.M.J., Zuilhof H. (2019). Romantic Surfaces: A Systematic Overview of Stable, Biospecific, and Antifouling Zwitterionic Surfaces. Langmuir.

[40] Gnanasampanthan T., Beyer C.D., Yu W., Karthäuser J.F., Wanka R., Spöllmann S., Becker H.W., Aldred N., Clare A.S., Rosenhahn A. (2021). Effect of Multilayer Termination on Nonspecific Protein Adsorption and Antifouling Activity of Alginate-Based Layer-by-Layer Coatings. Langmuir.

[41] Ladam G., Schaaf P., Decher G., Voegel J., Cuisinier F.J. (2002). Protein adsorption onto auto-assembled polyelectrolyte films. Biomol. Eng..

[42] Heuberger R., Sukhorukov G., Vörös J., Textor M., Möhwald H. (2005). Biofunctional polyelectrolyte multilayers and microcapsules: Control of nonspecific and bio-specific protein adsorption. Adv. Funct. Mater..

[43] Szczepanowicz K., Kruk T., Światek W., Bouzga A.M., Simon C.R., Warszyński P. (2018). Poly(l-glutamic acid)-g-poly(ethylene glycol) external layer in polyelectrolyte multilayer films: Characterization and resistance to serum protein adsorption. Colloids Surf. B Biointerfaces.

[44] Pasche S., De Paul S.M., Vörös J., Spencer N.D., Textor M. (2003). Poly (l-lysine)-g raft-poly (ethylene glycol) Assembled Monolayers on Niobium Oxide Surfaces: A Quantitative Study of the Influence of Polymer Interfacial Architecture on Resistance to Protein Adsorption by ToF-SIMS and in Situ OWLS. Langmuir.

[45] Sauerbrey G. (1959). Verwendung von Schwingquarzen zur Wägung dünner Schichten und zur Mikrowägung. Z. Phys..

[46] Giesbers M., Kleijn J.M., Stuart M.A.C. (2002). The electrical double layer on gold probed by electrokinetic and surface force measurements. J. Colloid Interface Sci..

[47] Gergely C., Bahi S., Szalontai B., Flores H., Schaaf P., Voegel J., Cuisinier F.J. (2004). Human serum albumin self-assembly on weak polyelectrolyte multilayer films structurally modified by pH changes. Langmuir.

[48] Greg T. (2013). Hermanson. Bioconjugate Techniques.

